# A four-locus phylogeny of rib-stiped cupulate species of *Helvella* (Helvellaceae, Pezizales) with discovery of three new species

**DOI:** 10.3897/mycokeys.60.38186

**Published:** 2019-10-31

**Authors:** Xin-Cun Wang, Tie-Zhi Liu, Shuang-Lin Chen, Yi Li, Wen-Ying Zhuang

**Affiliations:** 1 State Key Laboratory of Mycology, Institute of Microbiology, Chinese Academy of Sciences, Beijing 100101, China Institute of Microbiology, Chinese Academy of Sciences Beijing China; 2 College of Life Sciences, Chifeng University, Chifeng, Inner Mongolia 024000, China Chifeng University Chifeng China; 3 College of Life Sciences, Nanjing Normal University, Nanjing, Jiangsu 210023, China Nanjing Normal University Nanjing China; 4 College of Food Science and Engineering, Yangzhou University, Yangzhou, Jiangsu 225127, China Yangzhou University Yangzhou China

**Keywords:** Ascomycota, DNA barcode, phylogeny, taxonomy, typification

## Abstract

*Helvella* species are ascomycetous macrofungi with saddle-shaped or cupulate apothecia. They are distributed worldwide and play an important ecological role as ectomycorrhizal symbionts. A recent multi-locus phylogenetic study of the genus suggested that the cupulate group of *Helvella* was in need of comprehensive revision. In this study, all the specimens of cupulate *Helvella* sensu lato with ribbed stipes deposited in HMAS were examined morphologically and molecularly. A four-locus phylogeny was reconstructed using partial sequences of the heat shock protein 90, nuclear rDNA internal transcribed spacer region 2, nuclear large subunit ribosomal DNA and translation elongation factor 1-α genes. Three clades were revealed in *Helvella* sensu stricto. Twenty species were included in the analysis, of which 13 are distributed in China. Three new species, *H. acetabuloides*, *H. sichuanensis* and *H. tianshanensis*, are described and illustrated in detail. A neotype was designated for *H. taiyuanensis*. *Helvella calycina* is a new record for China, while *Dissingia leucomelaena* should be excluded from Chinese mycota. Hsp90 and ITS2 are recommended as useful supplementary barcodes for species identifications of the genus.

## Introduction

The genus *Helvella* L. contains a group of ascomycetous macrofungi with saddle-shaped or cupulate apothecia. *Helvella* species are distributed worldwide, especially in temperate regions (Dissing 1966, Abbott and Currah 1997). Some of them are edible, for example, *H. bachu* Q. Zhao, Zhu L. Yang & K.D. Hyde (Zhao et al. 2016a) and *H. taiyuanensis* B. Liu, Du & J.Z. Cao (Liu et al. 1985), and some are medicinal, for example, *H. lacunosa* Afzel. (Shameem et al. 2016). They are also important as ectomycorrhizal symbionts (Tedersoo et al. 2006, Healy et al. 2013, Hwang et al. 2015).

*Helvella* was established in 1753 and more than 400 names attributable to the genus have been recorded in the databases of Index Fungorum and MycoBank. Several taxonomic treatments were proposed, based on morphological characters (Table 1). Seven sections were established by Dissing (1966): sections *Acetabulum*, *Crispae*, *Elasticae*, *Ephippium*, *Lacunosae*, *Leucomelaenae* and *Macropodes*. Amongst them, the sections *Acetabulum* and *Leucomelaenae* included the species having cup-shaped apothecia with ribbed stipes. Similarly, six to eight infrageneric groups (sections or subgenus) were recognised by different authors (Weber 1972, Häffner 1987, Abbott and Currah 1997). Meanwhile, many additional species were added to the genus (Weber 1975, Harmaja 1976, 1977a, b, 1978, 1979, Abbott and Currah 1988). A checklist of cupulate *Helvella* species having ribbed stipes and their infrageneric positions are summarised in Table 2. Recently, *Helvella* sensu stricto was found to be associated with *Balsamia* Vittad., *Dissingia* K. Hansen, X.H. Wang & T. Schumach., *Midotis* Fr., *Pindara* Velen. and *Underwoodia* Peck in Helvellaceae (Hansen and Pfister 2006; Hansen et al. 2019). Amongst them, *Dissingia* was proposed to accommodate the species formerly placed in Helvella section Leucomelaenae (Hansen et al. 2019).

**Table 1.** Comparison of the taxonomic systems established in *Helvella*.

**Dissing (1966)**	**Weber (1972)**	**Häffner (1987)**	**Abbott and Currah (1997)**	**Hansen et al. (2019)**
Section Leucomelaenae Dissing	Section Leucomelaenae Dissing	Section Leucomelaenae Dissing	Subgenus Leucomelaenae (Dissing) S.P. Abbott	*Dissingia* K. Hansen, X.H. Wang & T. Schumach.
		Section Solitariae Häffner		*Helvella* L.
Section Acetabulum Dissing	Section Acetabulum Dissing	Section Acetabulum Dissing		
Section Crispae Dissing	Section Helvella L.	Section Helvella L.	Subgenus Helvella L.	
Section Lacunosae Dissing	Section Lacunosae Dissing	Section Lacunosae Dissing		
Section Elasticae Dissing	Section Elasticae Dissing	Section Elasticae Dissing	Subgenus Elasticae (Dissing) S.P. Abbott	
Section Ephippium Dissing	Section Ephippium Dissing	Section Ephippium Dissing		
Section Macropodes Dissing	Section Macropodes Dissing	Section Macropodes Dissing	Subgenus Macropodes (Dissing) S.P. Abbott	
			Subgenus Cupuliformes S.P. Abbott	
			Subgenus Silvicolae (S.P. Abbott) S.P. Abbott	*Midotis* Fr.

With the development of molecular phylogenetics, the taxonomy of *Helvella* has been re-evaluated. Sequences of nuclear large and small subunit ribosomal DNA (LSU and SSU) were adopted for phylogenetic inference of *Helvella* sensu lato and its allied genera (Hansen and Pfister 2006, Tedersoo et al. 2006, Laessoe and Hansen 2007). Protein-coding genes, RNA polymerase II the largest subunit (RPB1), the second largest subunit (RPB2) and translation elongation factor 1-α (TEF1) were also applied (Bonito et al. 2013, Hansen et al. 2013). Nguyen et al. (2013) explored *Helvella* phylogeny using large-scale sequence analysis of LSU and the nuclear rDNA internal transcribed spacer region (ITS) and reported two new species from North America based on molecular and morphological evidence. On the basis of examinations of the type specimens and LSU sequence analysis, Landeros et al. (2012, 2015) concluded that the sections *Elasticae*, *Helvella*, *Lacunosae* and *Leucomelaenae* were monophyletic. Skrede et al. (2017) studied molecular characteristics of 55 European species, described seven new species based on the sequence divergences of LSU, RPB2, TEF1 and heat shock protein 90 gene (Hsp90), and designated neotypes and epitypes for 30 of them. Five clades and 18 lineages were distinguished according to the phylogeny inferred from the combined Hsp90 and RPB2 datasets. The above work provides background information for understanding the species concept of *Helvella*. In their updated study, Hansen et al. (2019) defined *Helvella* s. s., treated the cupulate *H. leucomelaena* (Pers.) Nannf. lacking crozier at the ascus base as a separate genus *Dissingia*, retrieved the generic name *Pindara*, and transferred *H. aestivalis* (R. Heim & L. Rémy) Dissing & Raitv. to *Balsamia*. Brief comparisons amongst different taxonomic treatments are shown in Table 1.

In China, Teng (1963) recorded 11 species of *Helvella* and Tai (1979) listed 15 taxa. Liu, Cao and their collaborators (Liu et al. 1985, Liu and Cao 1988, Cao and Liu 1990, Cao et al. 1990) published nine species, new to the genus. With the additional investigations, our knowledge of the group accumulated (Zhuang 1989, 1995, 1996, 1997, 1998, Zhuang and Wang 1998a, 1998b, Yu et al. 2000, Wang and Chen 2002, Xu 2002, Zhuang 2004, Zhuang and Yang 2008). Zhuang et al. (2018) provided a checklist of 37 *Helvella* species occurring in China up to 2013. Recently, Zhao and his collaborators (Ariyawansa et al. 2015, Zhao et al. 2015, Hyde et al. 2016, Wang et al. 2016, Zhao et al. 2016a, 2016b, Tibpromma et al. 2017) described 12 new species with two bearing cupulate apothecia (Table 2), as well as two new Chinese records, *H. subglabra* N.S. Weber and *H. ulvinenii* Harmaja. There are about 51 species currently known from the country.

The present study is aimed at exploring species diversity of the cupulate *Helvella* species with ribbed stipes.

**Table 2.** A checklist of cupulate *Helvella* species sensu lato with ribbed stipes.

Species	Section Acetabulum	Section Leucomelaenae	Section Solitariae	Section Macropodes	Subgenus Leucomelaenae	Remark
*Acetabula calyx* Sacc., 1873	–	Syn. of *H. solitaria* (Dissing 1966); Syn. of *H. leucomelaena* (Harmaja 1977a)	–	–	Syn. of *H. leucomelaena* (Abbott and Currah 1997)	Syn. of *H. leucomelaena* (Landeros et al. 2015)
*Balsamia aestivalis* (R. Heim & L. Rémy) K. Hansen, Skrede & T. Schumach, 2019	–	Häffner 1987	–	–	Abbott and Currah 1997	as *Helvella aestivalis*
*Dissingia crassitunicata* (N.S. Weber) T. Schumach & Skrede, 2019	–	Weber 1975, Häffner 1987	–	–	Abbott and Currah 1997	as *Helvella crassitunicata*
*Dissingia confusa* (Harmaja) K. Hansen & X.H. Wang, 2019	–	Harmaja 1977a, Häffner 1987	–	–	Syn. of *H. leucomelaena* (Abbott and Currah 1997)	as *Helvella confusa*
*Dissingia leucomelaena* (Pers.) K. Hansen & X.H. Wang, 2019	–	Dissing 1966, Weber 1975, Häffner 1987	–	–	Abbott and Currah 1997	as *Helvella leucomelaena*
*Dissingia oblongispora* (Harmaja) T. Schumachand Skrede, 2019	–	Harmaja 1978, Häffner 1987	–	–	Abbott and Currah 1997	as *Helvella oblongispora*
*Helvella acetabulum* (L.) Quél, 1874	Dissing 1966, Weber 1972, Häffner 1987	–	–	–	Abbott and Currah 1997	Valid species
*Helvella arctoalpina* Harmaja, 1977	Harmaja 1977b, Häffner 1987	–	–	–	Syn. of *H. verruculosa* (Abbott and Currah 1997)	Valid species
*Helvella calycina* Skrede, T.A. Carlsen & T. Schumach, 2017	–	–	–	–	–	Valid species
*Helvella costata* Schwein, 1822	–	–	–	–	Syn. of *H. acetabulum* (Abbott and Currah 1997)	Valid species
*Helvella costifera* Nannf, 1953	Dissing 1966, Häffner 1987	–	–	–	Abbott and Currah 1997	Valid species
*Helvella dryadophila* Harmaja, 1977	Harmaja 1977b, Häffner 1987	–	–	–	Syn. of *H. verruculosa* (Abbott and Currah 1997)	Valid species
*Helvella floriforma* Q. Zhao & K.D. Hyde, 2016^*^	–	–	–	–	–	Valid species
*Helvella griseoalba* N.S. Weber, 1972	Weber 1972, Häffner 1987	–	–	–	Syn. of *H. costifera* (Abbott and Currah 1997)	Valid species
*Helvella helvellula* (Durieu) Dissing, 1966	–	Dissing 1966	–	–	–	Member of lasunosa clade (Skrede et al. 2017)
*Helvella hyperborea* Harmaja, 1978	Harmaja 1978, Häffner 1987	–	–	–	Abbott and Currah 1997	Valid species
*Helvella jiaohensis* J.Z. Cao, L. Fan & B. Liu, 1990^*^	–	–	–	–	–	Holotype lost
*Helvella jilinensis* J.Z. Cao, L. Fan & B. Liu, 1990^*^	–	–	–	–	–	Holotype lost
*Helvella pedunculata* Harmaja, 1978	–	Harmaja 1978, Häffner 1987	–	–	Syn. of *H. leucomelaena* (Abbott and Currah 1997)	?Syn. of *H. costifera* (Skrede et al. 2017)
*Helvella pocillum* Harmaja, 1976	Häffner 1987	Harmaja 1976	–	–	–	Syn. of *B. aestivalis* (Hansen et al. 2019)
*Helvella queletii* Bres, 1882	–	Syn. of *H. solitaria* (Harmaja 1977a, Häffner 1987)	–	Dissing 1966, Weber 1972	Syn. of *H. solitaria* (Abbott and Currah 1997)	Syn. of *H. solitaria* (Landeros et al. 2012)
*Helvella robusta* S.P. Abbott, 1988	Abbott and Currah 1988	–	–	–	Abbott and Currah 1997	Valid species
*Helvella solitaria* P. Karst, 1871	–	Dissing 1966	Häffner 1987	–	Abbott and Currah 1997	Valid species
*Helvella taiyuanensis* B. Liu, Du & J.Z. Cao, 1985^*^	–	–	–	–	–	Neotypification here
*Helvella tinta* Q. Zhao, B. Feng & K.D. Hyde, 2016^*^	–	–	–	–	–	Valid species
*Helvella ulvinenii* Harmaja, 1979	Harmaja 1979	–	Häffner 1987	–	Abbott and Currah 1997	Syn. of *H. solitaria* (Landeros et al. 2015)
*Helvella unicolor* (Boud.) Dissing, 1966	Dissing 1966, Häffner 1987	–	–	–	Abbott and Currah 1997	In need of reassessment (Skrede et al. 2017)
*Helvella verruculosa* (Sacc.) Harmaja, 1978	–	–	–	–	Abbott and Currah 1997	In need of reassessment (Skrede et al. 2017)

Syn.: synonym; ^*^ indicates the species originally described from China.

## Materials and methods

### Fungal materials and morphological observations

Collections of the cupulate *Helvella* species with ribbed stipes, deposited in the Herbarium Mycologicum Academiae Sinicae (**HMAS**), were re-examined, including those originally deposited in the Mycological Herbarium of Shanxi University (**MHSU**). Specimens recently collected from Beijing, Inner Mongolia, Hubei and Sichuan provinces were identified (Table 3). Morphological observations were conducted following Wang and Zhuang (2019). In measurements, Q refers to length/width ratio of ascospores for which the medians are given.

**Table 3.** Fungal species and sequences used in phylogenetic analyses.

Species	Voucher	Locality	*HSP90*	ITS	LSU	*TEF1*	Label	Reference
*Balsamia aestivalis* (R. Heim & L. Rémy) K. Hansen, Skrede & T. Schumach.	KH.10.133	Sweden	–	–	MK100250	MK113869	*Balsamia aestivalis*	Hansen et al. 2019
	O-253217	Norway	–	–	MK100251	MK113870	*Balsamia aestivalis*	Hansen et al. 2019
*Balsamia platyspora* Berk.	TUR206101	Finland	–	–	MK100252	MK113871	*Balsamia platyspora*	Hansen et al. 2019
*Dissingia confusa* (Harmaja) K. Hansen & X.H. Wang	H437^*^	Norway	KY784529	–	KY773164	–	*Helvella confusa*	Skrede et al. 2017
	HMAS 27728^*^	Qinghai, China	**MK652180**	**MK592119**	–	–	*Helvella confusa*	This study
	HMAS 38328^*^	Xinjiang, China	**MK652181**	**MK592120**	–	–	*Acetabula leucomelas*	This study
*Dissingia crassitunicata* (N.S. Weber) T. Schumach. & Skrede	H222^*^	Canada	KY784342	–	KY773053	–	*Helvella crassitunicata*	Skrede et al. 2017
*Dissingia leucomelaena* (Pers.) K. Hansen & X.H. Wang	H404, epitype	Sweden	KY784500	–	–	–	*Helvella leucomelaena*	Skrede et al. 2017
	H115^*^	USA	KY784253	–	KY772970	–	*Helvella leucomelaena*	Skrede et al. 2017
	KH.06.01 = H115	USA	–	–	KC012682	KC109207	*Helvella leucomelaena*	Hansen et al. 2013
	He273	Australia	–	–	JX993075	–	*Helvella leucomelaena*	Landeros et al. 2015
	He286, isotype	Italy	–	–	JX993051	–	*Acetabula calyx*	Landeros et al. 2015
	HMAS 61351	Denmark	**MK652201**	–	–	–	*Helvella leucomelaena*	This study
	HMAS 61356^*^	Sweden	**MK652202**	**MK592137**	–	–	*Helvella leucomelaena*	This study
*Dissingia oblongispora* (Harmaja) T. Schumach. & Skrede	H132^*^	Norway	KY784265	–	KY772983	–	*Helvella oblongispora*	Skrede et al. 2017
	HMAS 38329^*^	Xinjiang, China	**MK652203**	**MK592138**	–	–	*Helvella acetabulum*	This study
	HMAS 74657^*^	Gansu, China	**MK652204**	**MK592139**	–	–	*Helvella leucomelaena*	This study
	HMAS 75147^*^	Sichuan, China	**MK652205**	**MK592140**	–	**MK652162**	*Helvella leucomelaena*	This study
	HMAS 75151	Sichuan, China	**MK652206**	**MK592141**	–	–	*Helvella leucomelaena*	This study
	HMAS 75183	Sichuan, China	**MK652207**	**MK592142**	–	–	*Helvella leucomelaena*	This study
	HMAS 75960	Sichuan, China	**MK652208**	**MK592143**	–	–	*Helvella cupuliformis*	This study
	HMAS 86050	Xinjiang, China	–	**MK592144**	–	–	*Helvella acetabulum*	This study
	HMAS 86051	Xinjiang, China	–	**MK592145**	–	**MK652163**	*Helvella acetabulum*	This study
	HMAS 86160	Shanxi, China	–	**MK592146**	–	–	*Helvella leucomelaena*	This study
*Helvella acetabuloides* X.C. Wang & W.Y. Zhuang	HMAS 279703^*^, CFSZ 2044, holotype	Inner Mongolia, China	**MK652219**	**MK592155**	–	**MK652168**	*Helvella acetabulum*	This study
	HMAS 23842^*^	Shaanxi, China	**MK652220**	–	–	–	*Acetabula vulgaris*	This study
*Helvella acetabulum* (L.) Quél.	H410, epitype	Sweden	KY784506	–	KY773154	–	*Helvella acetabulum*	Skrede et al. 2017
	H133^*^	Norway	KY784266	–	KY772984	KY772875	*Helvella acetabulum*	Skrede et al. 2017
	HMAS 7046^*^	Czech	**MK652177**	**MK592116**	–	–	*Acetabula vulgaris*	This study
	HMAS 61353	Denmark	**MK652176**	–	–	–	*Helvella acetabulum*	This study
	HMAS 243823^*^	UK	**MK652174**	**MK592114**	**MK592099**	–	*Helvella acetabulum*	This study
	HMAS 23839	Qinghai, China	**MK652171**	**MK592112**	–	–	*Helvella acetabulum*	This study
	HMAS 23841	Beijing, China	**MK652172**	**MK592113**	–	–	*Helvella acetabulum*	This study
	HMAS 23843	Qinghai, China	**MK652173**	–	–	–	*Acetabula vulgaris*	This study
	HMAS 38129	Xinjiang, China	**MK652175**	**MK592115**	–	–	*Helvella acetabulum*	This study
*Helvella acetabulum* (L.) Quél.	HMAS 75176^*^	Sichuan, China	**MK652178**	**MK592117**	–	**MK652156**	*Helvella acetabulum*	This study
*Helvella arctoalpina* Harmaja	H293, holotype	Norway	KY784406	–	–	–	*Helvella arctoalpina*	Skrede et al. 2017
	H033^*^	Norway	KY784207	–	KY772924	KY772841	*Helvella arctoalpina*	Skrede et al. 2017
*Helvella calycina* Skrede, T.A. Carlsen & T. Schumach.	H022^*^, epitype	Norway	KY784198	–	KY772915	KY772833	*Helvella calycina*	Skrede et al. 2017
	HMAS 279704^*^, CFSZ 2658	Inner Mongolia, China	**MK652179**	**MK592118**	**MK592100**	**MK652157**	*Helvella acetabulum*	This study
*Helvella costata* Schwein.	H100^*^	USA	KY784244	–	KY772962	–	*Helvella costata*	Skrede et al. 2017
*Helvella costifera* Nannf.	H298, epitype	Sweden	KY784409	–	–	–	*Helvella costifera*	Skrede et al. 2017
	H131^*^	Norway	KY784264	–	KY772982	KY772874	*Helvella costifera*	Skrede et al. 2017
	HMAS 61361	Shanxi, China	**MK652185**	–	–	–	*Helvella acetabulum*	This study
	HMAS 71778	Beijing, China	**MK652186**	**MK592124**	–	–	*Helvella costifera*	This study
	HMAS 83510	Xinjiang, China	**MK652187**	**MK592125**	–	–	*Helvella costifera*	This study
	HMAS 88497	Shanxi, China	**MK652188**	**MK592126**	–	–	*Helvella acetabulum*	This study
	HMAS 139024^*^	Shaanxi, China	**MK652182**	**MK592121**	**MK592101**	–	*Helvella* sp.	This study
	HMAS 187120^*^	Beijing, China	**MK652183**	**MK592122**	**MK592102**	**MK652158**	*Helvella* sp.	This study
	HMAS 280301^*^	Yunnan, China	**MK652184**	**MK592123**	**MK592103**	**MK652159**	*Helvella* sp.	This study
*Helvella dryadophila* Harmaja	H302, holotype	Norway	KY784412	–	–	–	*Helvella dryadophila*	Skrede et al. 2017
	H180^*^	Norway	KY784309	–	KY773024	KY772883	*Helvella dryadophila*	Skrede et al. 2017
*Helvella floriforma* Q. Zhao & K.D. Hyde	HKAS 90224, Holotype	Yunnan, China	–	–	KX239771	–	*Helvella floriforma*	Hyde et al. 2016
*Helvella griseoalba* N.S. Weber	He164, holotype	USA	–	–	JX993066	–	*Helvella griseoalba*	Landeros et al. 2015
	H306^*^	USA	KY784416	–	–	–	*Helvella griseoalba*	Skrede et al. 2017
*Helvella hyperborea* Harmaja	H491^*^	Finland	KY784569	–	–	–	*Helvella hyperborea*	Skrede et al. 2017
	HMAS 23840	Gansu, China	**MK652189**	–	–	–	*Helvella acetabulum*	This study
	HMAS 38331	Xinjiang, China	**MK652190**	–	–	–	*Helvella costifera*	This study
	HMAS 83506	Xinjiang, China	**MK652191**	**MK592127**	–	–	*Helvella costifera*	This study
	HMAS 83507	Xinjiang, China	**MK652192**	**MK592128**	–	–	*Helvella costifera*	This study
	HMAS 83508	Xinjiang, China	**MK652193**	**MK592129**	–	–	*Helvella costifera*	This study
	HMAS 83509	Xinjiang, China	**MK652194**	**MK592130**	–	–	*Helvella costifera*	This study
	HMAS 83511	Xinjiang, China	**MK652195**	**MK592131**	–	**MK652160**	*Helvella costifera*	This study
*Helvella hyperborea* Harmaja	HMAS 83512	Xinjiang, China	**MK652196**	**MK592132**	–	–	*Helvella costifera*	This study
	HMAS 85476	Xinjiang, China	**MK652197**	**MK592133**	–	–	*Helvella acetabulum*	This study
	HMAS 85591^*^	Shanxi, China	**MK652198**	**MK592134**	–	–	*Helvella leucomelaena*	This study
	HMAS 85673^*^	Shanxi, China	**MK652199**	**MK592135**	–	–	*Helvella solitaria*	This study
	HMAS 86043^*^	Xinjiang, China	**MK652200**	**MK592136**	–	**MK652161**	*Helvella costifera*	This study
*Helvella robusta* S.P. Abbott	He163, holotype	Canada	–	–	JX993079	–	*Helvella robusta*	Landeros et al. 2015
*Helvella sichuanensis* X.C. Wang & W.Y. Zhuang	10706^*^, HMAS 254610, holotype	Sichuan, China	**MK652221**	**MK592156**	**MK592107**	**MK652169**		This study
*Helvella solitaria* P. Karst.	H370, epitype	Sweden	KY784470	–	–	–	*Helvella solitaria*	Skrede et al. 2017
	H004^*^	Norway	KY784184	–	KY772902	KY772819	*Helvella solitaria*	Skrede et al. 2017
	He248, holotype	Finland	–	–	JX993085	–	*Helvella ulvinenii*	Landeros et al. 2015
	HMAS 41140^*^	Netherlands	**MK652211**	**MK592148**	–	–	*Helvella queletii*	This study
	HMAS 58371	Czech	**MK652212**	–	–	–	*Helvella queletii*	This study
	HMAS 27727^*^	Qinghai, China	**MK652209**	**MK592147**	–	–	*Helvella confusa*	This study
	HMAS 27951	Jilin, China	**MK652210**	–	–	–	*Helvella confusa*	This study
	HMAS 73509	Sichuan, China	**MK652213**	**MK592149**	–	–	*Helvella acetabulum*	This study
	HMAS 75175^*^	Sichuan, China	**MK652214**	**MK592150**	–	**MK652164**	*Helvella leucomelaena*	This study
*Helvella taiyuanensis* B. Liu, Du & J.Z. Cao	HMAS 85689^*^, neotype	Shanxi, China	**MK652217**	**MK592153**	–	–	*Helvella taiyuanensis*	This study
	HMAS 277500^*^	Yunnan, China	**MK652216**	**MK592152**	**MK592105**	**MK652166**	*Helvella* sp.	This study
	11925^*^, HMAS 254611	Beijing, China	**MK652215**	**MK592151**	**MK592104**	**MK652165**		This study
	MCCNNU 6499^*^, HMAS 279702	Hubei, China	**MK652218**	**MK592154**	**MK592106**	**MK652167**	*Helvella solitaria*	This study
*Helvella tianshanensis* X.C. Wang & W.Y. Zhuang	HMAS 86040^*^, holotype	Xinjiang, China	**MK652222**	**MK592157**	**MK592108**	**MK652170**	*Helvella costifera*	This study
	HMAS 88611^*^	Xinjiang, China	**MK652223**	**MK592158**	–	–	*Helvella acetabulum*	This study
*Helvella tinta* Q. Zhao, B. Feng & K.D. Hyde	HKAS 82560, holotype	Sichuan, China	–	KX239842	KX239772	–	*Helvella tinta*	Hyde et al. 2016
*Helvella crispa* (Scop.) Fr.	H408^*^, epitype	Sweden	KY784504	–	–	–	*Helvella crispa*	Skrede et al. 2017
	H135	Norway	KY784268	–	KY772986	–	*Helvella crispa*	Skrede et al. 2017
	HKAS 75434	Germany	–	JX462572	KR493479	KT254487	*Helvella crispa*	Zhao et al. 2015
*Helvella elastica* Bull.	H066^*^	Sweden	KY784230	–	KY772950	KY772858	*Helvella elastica*	Skrede et al. 2017
*Helvella lacunosa* Afzel.	H407, epitype	Sweden	KY784503	–	KY773152	–	*Helvella lacunosa*	Skrede et al. 2017
	H039^*^	Norway	KY784213	–	KY772930	KY772845	*Helvella lacunosa*	Skrede et al. 2017
*Helvella macropus* (Pers.) P. Karst.	H412, epitype	Sweden	KY784507	–	–	–	*Helvella macropus*	Skrede et al. 2017
	H073^*^	Norway	KY784233	–	KY772954	KY772863	*Helvella macropus*	Skrede et al. 2017
*Midotis lingua* Fr.	H283^*^	Switzerland	KY784397	–	KY773093	–	*Wynnella silvicola*	Skrede et al. 2017
	HMAS 67962^*^	Germany	**MK652224**	**MK592159**	**MK592109**	–	*Wynnella auricula*	This study
	HMAS 71896^*^	Shanxi, China	**MK652225**	**MK592160**	**MK592110**	–	*Wynnella silvicola*	This study
	HMAS 74656	Gansu, China	**MK652226**	**MK592161**	**MK592111**	–	*Helvella silvicola*	This study
	HMAS 83548	Xinjiang, China	**MK652227**	**MK592162**	–	–	*Wynnella auricula*	This study
*Pindara terrestris* Velen.	KH.12.67	Sweden	–	–	MK100279	MK113889	*Pindara terrestris*	Hansen et al. 2019
	S-F327988	Sweden	–	–	MK100280	MK113896	*Pindara terrestris*	Hansen et al. 2019
	T. Kekki 168	Finland	–	–	MK100281	MK113897	*Pindara terrestris*	Hansen et al. 2019
*Underwoodia columnaris* Peck	Kanouse 1951	USA	–	–	U42685	–	*Underwoodia columnaris*	O'Donnell et al. 1997

^*^ Taxa included in the four-locus sequence analysis; Note: GenBank accession numbers in bold indicating the newly generated sequences.

### DNA extraction, PCR amplification and sequencing

Well-preserved specimens were selected for DNA extraction using a Plant Genomic DNA Kit (DP305, TIANGEN Biotech, Beijing, China). Partial Hsp90, ITS2, LSU and TEF1 were amplified by PCR using primers H_hspf and H_hspr (Skrede et al. 2017), ITS3 and ITS4 (White et al. 1990), LROR and LR5 (Vilgalys and Hester 1990) and EF1-983F and EF1-1567R (Rehner and Buckley 2005). Products were sequenced on an ABI 3730 DNA Sequencer (Applied Biosystems).

### Phylogenetic analyses

Sequences obtained from this study and those retrieved from GenBank are listed in Table 3. Four single gene datasets and two combined datasets were compiled. Sequences were aligned using MAFFT 7.221 (Katoh and Standley 2013) and subsequently processed with BioEdit 7.1.10 (Hall 1999). A Maximum-Likelihood (ML) tree for each single gene data was generated using MEGA 6.0.6 (Tamura et al. 2013) with the most suitable nucleotide substitution model and 1,000 replicates of bootstrap (BP) tests. For the combined four-gene dataset, the ML tree was determined using RAxML-HPC2 on XSEDE 8.2.12 on CIPRES Science Gateway (Miller et al. 2010) with the default GTRCAT model. Bayesian Inference (BI) analysis was performed with MrBayes 3.2.6 (Ronquist et al. 2012) using a Markov Chain Monte Carlo (MCMC) algorithm. Appropriate nucleotide substitution models and parameters were determined via ModelTest 3.7 (Posada and Crandall 1998). The first 25% of the trees were excluded as the burn-in phase and posterior probability (PP) values were estimated with the remaining 75% of trees. *Helvella crispa* (Scop.) Fr., *H. elastica* Bull., *H. lacunosa* Afzel. and *H. macropus* (Pers.) P. Karst. are the representatives of the formerly recognised sections *Crispae*, *Elasticae*, *Lacunosae* and *Macropodes*, respectively. *Midotis lingua* Fr. served as the outgroup taxon of the four-gene phylogeny and *Underwoodia columnaris* Peck worked for the two-gene analysis.

## Results

Fifty-one specimens of the rib-stiped cupulate species of *Helvella* s. l. deposited in HMAS and five recent collections were examined. A total of 125 sequences of the *Helvella* and *Dissingia* samples and 11 of the outgroup taxa were submitted to GenBank (Table 3).

**Figure 1.**
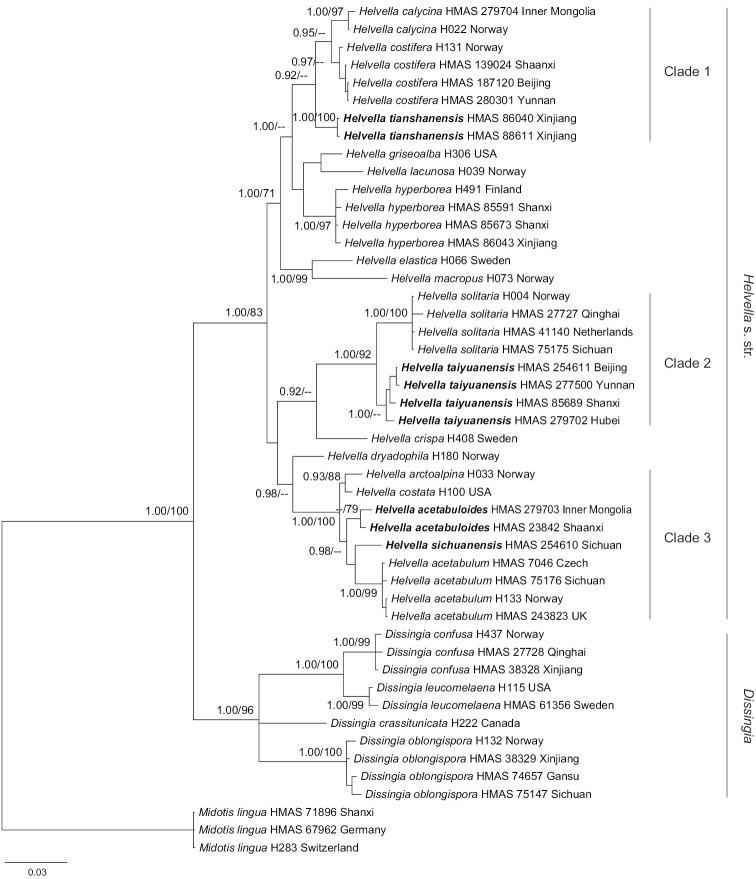
Bayesian phylogenetic tree of *Helvella* and *Dissingia* inferred from combined Hsp90, ITS2, LSU and TEF1 dataset. Posterior probability values ≥ 0.90 (left) and bootstrap values ≥ 70% (right) are indicated at nodes.

The combined four-locus dataset included 48 taxa of *Helvella* s. s. and *Dissingia* in an alignment of 1788 bp, including 236 bp of Hsp90, 348 bp of ITS2, 690 bp of LSU and 514 bp of TEF1. Kimura 2-parameter (K2) with gamma distribution (+G) was determined as the most suitable model for ML analysis. Tamura-Nei with gamma distribution and invariant sites (TrN+I+G) was selected by Akaike Information Criterion as the best fit for the BI analysis. As shown in Figure 1, three clades and some independent lineages were recognised amongst the cupulate taxa of *Helvella* s. s. Clade 1 consisted of *H. calycina*, *H. costifera* and *H. tianshanensis*; Clade 2 included *H. solitaria* and *H. taiyuanensis*; and Clade 3 contained *H. acetabuloides*, *H. acetabulum*, *H. arctoalpina*, *H. costata* and *H. sichuanensis*. *Helvella dryadophila*, as an independent lineage, was sister to Clade 3, which was not supported by two of the single gene analyses (Suppl. material 1: Figures S1 and S4). *Helvella griseoalba* and *H. hyperborea* were situated outside the clades in all analyses.

**Figure 2.**
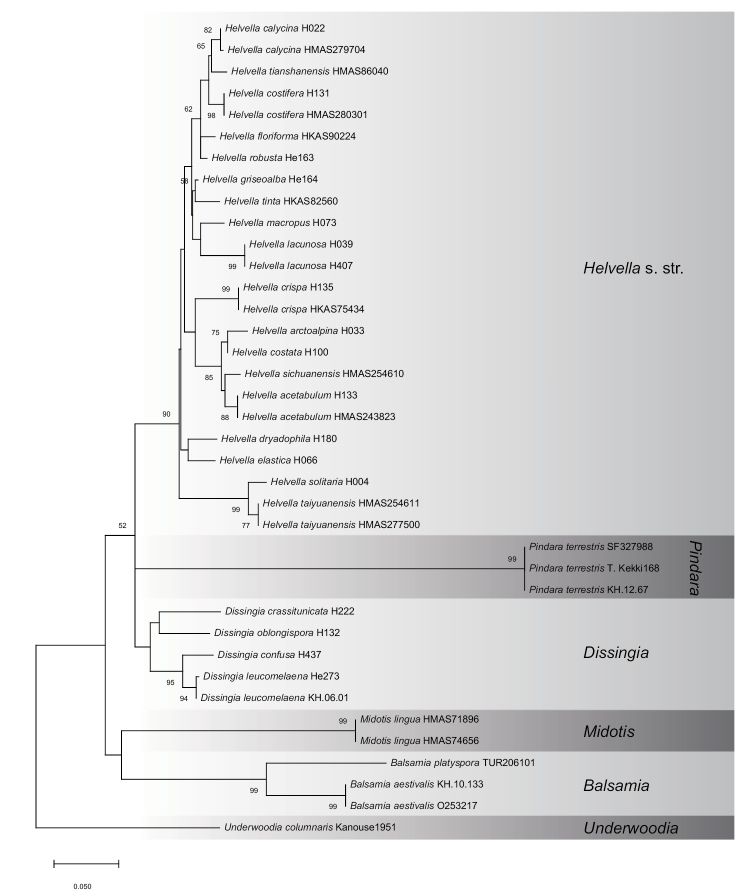
Maximum likelihood phylogeny of Helvellaceae inferred from combined LSU and TEF1 dataset. Bootstrap values ≥ 50% are indicated at nodes.

The combined LSU and TEF1 dataset was comprised of 38 taxa of *Balsamia*, *Dissingia*, *Helvella*, *Midotis*, *Pindara* and *Underwoodia*. The alignment is of 1239 bp, including 711 bp of LSU and 528 bp of TEF1. Tamura-Nei with gamma distribution (TN93+G) was determined as the most suitable model for ML analysis. Clades 1–3 were supported and *H. dryadophila* was outside Clade 3 (Figure 2), which are congruent with the four-gene analysis (Figure 1).

The Hsp90 dataset consisted of 84 sequences of *Helvella* and *Dissingia*. K2+G was determined as the most suitable model for ML analysis. Clades 2 and 3 were monophyletic, but Clade 1 was poorly supported (Suppl. material 1: Figure S1). The positions of the three undescribed species were consistent with that of the four-locus phylogeny.

The ITS2 dataset possessed 53 taxa of *Helvella* and *Dissingia*. Tamura 3-parameter with gamma distribution (T92+G) was determined as the most suitable model for ML analysis. Clades 1–3 were strongly supported. *Helvella tinta*, excluded from these clades, appeared to be sister of *H. hyperborea* (Suppl. material 1: Figure S2).

The LSU dataset comprised 40 sequences of *Helvella* and *Dissingia*. TN93+G was determined as the most suitable model for ML analysis. Clades 1–3 of *Helvella* were monophyletic, in which *H. floriforma* and *H. robusta*, absent in other trees, were located. *Dissingia* seemed to be not monophyletic (Suppl. material 1: Figure S3).

The TEF1 dataset consisted of 26 taxa of *Helvella* and *Dissingia*. K2+G was determined as the most suitable model for ML analysis. Clades 1–3 of *Helvella* were strongly supported (Suppl. material 1: Figure S4) and the phylogenetic positions of the three undescribed species recalled that of the multigene phylogeny (Figure 1).

## Taxonomy

### New species

#### Helvella acetabuloides

Taxon classificationFungiPezizalesHelvellaceae

X.C. Wang & W.Y. Zhuang sp. nov.

325488B8-66BA-5A9C-9FFE-655038853D58

Figure 3a–d

##### Holotype.

CHINA. Inner Mongolia Autonomous Region, Chifeng City, Harqin Banner, Shijia Town, Toudaoyingzi Village, 41°53'20"N, 119°1'1"E, on the ground under *Ostryopsis davidiana* Decne., 8 Aug 2002, T.Z. Liu & T.H. Liu, HMAS 279703 (= CFSZ 2044).

##### Etymology.

The species epithet refers to its similarity to *H. acetabulum*.

##### Description.

Apothecia stipitate to subsessile, cupulate, margin undulate, involute or revolute, 2.2–4.8 cm high and 2.5–4 cm diam. when dry; hymenium dull brown to reddish-brown when dry, receptacle surface light brown to brown when dry, glabrous; stipe terete or flattened, buff, light yellowish-brown to brown, surface ribbed, 0.5–3 × 0.4–1.3 cm, typically fluted with sharp-edged or rarely blunt ribs, ribs branching at the upper half of receptacle surface, reaching to the edge or ending 1–2 mm from the edge. Ectal excipulum of *textura angularis*, 75–100 µm thick, cells hyaline, outer cells arranged in chains, 16–21.5 × 7–8 µm. Medullary excipulum of *textura intricata*, 180–220 µm thick, hyphae hyaline. Asci subcylindrical, tapering and with crozier at base, 8-spored, 235–280 × 15–20 µm. Paraphyses filiform with apical portion very slightly enlarged, septate, hyaline, 4.5–5.5 µm wide at apex and 4–4.5 µm below. Ascospores ellipsoidal, hyaline, smooth, uniguttulate, 14–20 × 10–14.5 µm, median 16.2 × 12.3 µm, Q = 1.2–1.55, median 1.375, n = 50.

##### Additional specimen examined.

CHINA. Shaanxi Province, Baoji City, Taibai County, Mt. Taibai, 34°1'53"N, 107°25'33"E, alt. 2270 m, on the ground in broad-leaf forest, 26 Jun 1958, J.H. Yu 106, HMAS 23842.

##### Notes.

*Helvella acetabuloides* is nested with *H. acetabulum*, *H. arctoalpina*, *H. costata* and *H. sichuanensis* in Clade 3 (Figure 1). Its hymenium is reddish-brown when dry and different from that of *H. acetabulum* (brown when dry) and those of *H. arctoalpina* and *H. sichuanensis* (black when dry, Harmaja 1977b). The two specimens cited are identical in sequences of *Hsp90*. *Helvella acetabuloides* differs from *H. acetabulum* in 6 bp for Hsp90 (H410, epitype), 14 bp for ITS2 (HMAS 243823) and 17 bp for TEF1 (H133). It is distinguished from *H. arctoalpina* in 2 bp of Hsp90 (H293, holotype) and 11 bp of TEF1 (H033), from *H. costata* in 3 bp of Hsp90. It differs from *H. sichuanensis* in 1 bp of Hsp90, 20 bp of ITS2 and 11 bp of TEF1. PCR amplification of LSU failed.

**Figure 3.**
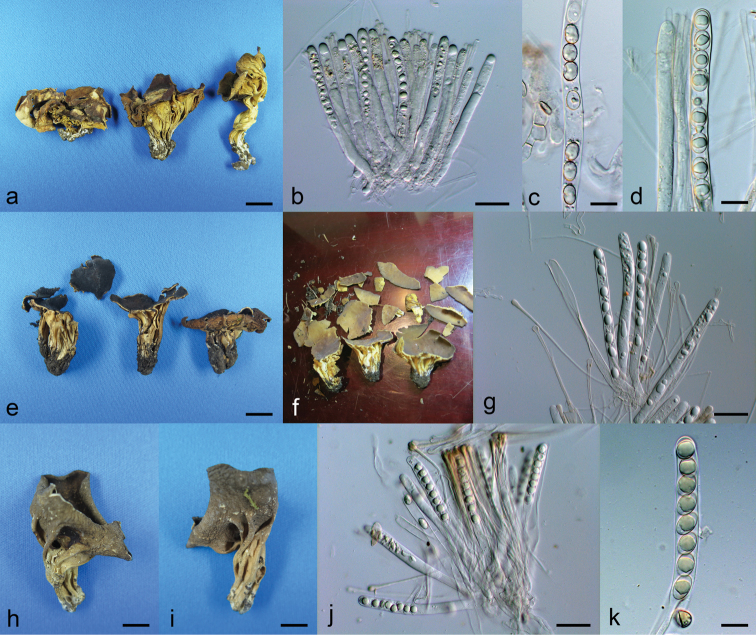
**a–d***Helvella acetabuloides*: **a** mature apothecia when dry (CFSZ 2044) **b** asci (HMAS 23842) **c, d** ascospores in ascus (c: CFSZ 2044, d: HMAS 23842) **e–g***Helvella sichuanensis* (HMAS 254610): **e** mature apothecia when dry **f** mature apothecia when fresh **g** ascospores in asci **h–k***Helvella tianshanensis* (HMAS 86040): **h, i** Mature apothecium when dry **j** asci **k** ascospores in ascus. Scale bars: 1 cm (**a, e**); 0.75 cm (**h, i**); 50 μm (**b, g, j**); 20 μm (**c, d, k**).

#### Helvella sichuanensis

Taxon classificationFungiPezizalesHelvellaceae

X.C. Wang & W.Y. Zhuang sp. nov.

B26640F5-749E-5827-9B98-508F5CAF8DE7

Figure 3e–g

##### Holotype.

CHINA. Sichuan Province, Garzê Tibetan Autonomous Prefecture, Daocheng County, Yading National Nature Reserve, 28°25'6"N, 100°21'26"E, alt. 3900 m, on the ground of mixed forest, 18 Aug 2016, J.P. Wang & X.C. Wang 10706, HMAS 254610.

##### Etymology.

The species epithet refers to the type locality of the fungus.

##### Description.

Apothecia stipitate, shallow-cupulate, margin entire and flattened when fresh, undulate, involute or revolute when dry, 5–6 cm diam. when fresh and 2.5–3.5 cm high when dry; hymenium yellowish-brown when fresh, nearly black when dry, receptacle surface buff to light brown when fresh, light brown to dark brown when dry, glabrous; stipe terete or flattened, buff to light brown, surface ribbed, 2.5–3 × 1.5–3 cm when fresh, 2–2.5 × 0.5–1.5 cm when dry, typically fluted with sharp-edged or rarely blunt ribs, ribs branching at the upper half of receptacle surface, reaching to the edge or ending 3–5 mm from the edge. Ectal excipulum of *textura angularis*, 100–180 µm thick, cells hyaline to light brown, outer cells 15–45 × 9–35 µm. Medullary excipulum of *textura intricata*, 300–500 µm thick, hyphae hyaline. Asci subcylindrical, tapering and with crozier at base, 8-spored, 225–325 × 13–18.5 µm. Paraphyses filiform with apical portion obviously swollen, septate, hyaline to light brown, 7–10.5 µm wide at apex and 3–4.5 µm below. Ascospores ellipsoidal, hyaline, smooth, uniguttulate, 15.5–18.5 × 10–12.5 µm, median 16.9 × 11.2 µm, Q = 1.3–1.7, median 1.48, n = 40.

##### Notes.

*Helvella sichuanensis* belongs to Clade 3 (Figure 1). Its hymenium is nearly black when dry, which is similar to that of *H. arctoalpina*, but different from those in *H. acetabulum* (brown when dry) and *H. acetabuloides* (reddish-brown when dry). When fresh, the hymenium is yellowish-brown, while that of *H. arctoalpina* is brown. Molecularly, it differs from *H. acetabulum* in 7 bp of Hsp90 (H410, epitype), 14 bp of ITS2 (HMAS 243823), 17 bp of LSU (H133) and 15 bp of TEF1 (H133); from *H. arctoalpina* in 1 bp of Hsp90 (H293, holotype), 25 bp of LSU (H033) and 11 bp of TEF1 (H033); and from *H. costata* in 2 bp of Hsp90 and 13 bp of LSU. The sequence divergences between *H. sichuanensis* and *H. acetabuloides* are 1 bp of Hsp90, 20 bp of ITS2 and 12 bp of TEF1.

#### Helvella tianshanensis

Taxon classificationFungiPezizalesHelvellaceae

X.C. Wang & W.Y. Zhuang sp. nov.

09A48BF3-ECA7-5100-9576-E424981BD683

Figure 3h–k

##### Holotype.

CHINA. Xinjiang Uygur Autonomous Region, Changji Hui Autonomous Prefecture, Jimsar County, 43°59'44"N, 89°10'31"E, alt. 1700 m, on the ground, 31 Jul 2003, W.Y. Zhuang & Y. Nong 4661, HMAS 86040.

##### Etymology.

The species epithet refers to the type locality of the fungus.

##### Description.

Apothecia stipitate, cupulate, margin undulate, involute, 2.5–3.5 cm high and 2–3 cm diam. when dry; hymenium greyish-brown, brown to dark brown, receptacle surface yellowish-brown to brown; stipe terete or flattened, buff, yellowish-brown, orange brown to brown, surface ribbed, 2–2.5 × 0.5–1.3 cm, typically fluted with rarely blunt ribs, ribs branching at the upper half of receptacle surface, reaching to the edge or ending 3–12 mm from the edge. Ectal excipulum of *textura angularis*, 120–150 µm thick, hyphae hyaline, outer cells 35–40 × 20–40 µm. Medullary excipulum of *textura intricata*, 350–600 µm thick, hyphae hyaline. Asci subcylindrical, tapering and with crozier at base, 8-spored, 240–275 × 12–24 µm. Paraphyses filiform, slightly enlarged at apical portion, septate, hyaline to light brown, 6–7.5 µm wide at apex and 3–4.5 µm below. Ascospores ellipsoidal, hyaline, smooth, uniguttulate, 17–21 × 11.5–13.5 µm, median 18.8 × 12.3 µm, Q = 1.35–1.7, median 1.51, n = 30.

##### Additional specimen examined.

CHINA. Xinjiang Uygur Autonomous Region, Urumqi City, Urumqi County, 43°28'47"N, 87°27'27"E, 12 Aug 1985, L. Fan & K. Tao 161, HMAS 88611.

##### Notes.

*Helvella tianshanensis* nested with *H. calycina* and *H. costifera* in Clade 1 (Figure 1). These three species are hardly separated by gross morphology and anatomic structures. *Helvella tianshanensis* differs from *H. calycina* in 4 bp of Hsp90 (H022, epitype), 16 bp of ITS2 (HMAS 279704), 9 bp of LSU (H022) and 15 bp of TEF1 (H022); and it is different from *H. costifera* in 3 bp of Hsp90 (H298, epitype), 12 bp of ITS2 (HMAS 187120), 11 bp of LSU (H131) and 13 bp of TEF1 (H131). The two specimens of the new species are identical in Hsp90 and ITS2.

### New Chinese record

#### Helvella calycina

Taxon classificationFungiPezizalesHelvellaceae

Skrede, T.A. Carlsen & T. Schumach., Persoonia 39: 221, 2017

FA734B9A-7D79-59C4-A722-A3FFE6D51D54

##### Specimen examined.

CHINA. Inner Mongolia Autonomous Region, Xilingol League, Zhenglan Banner, Yihehaierhan Sumu, 42°23'8"N, 116°10'17"E, 21 August 2005, on the ground, T.Z. Liu & X.L. Bai, HMAS 279704 (= CFSZ 2658).

##### Notes.

*Helvella calycina* is a new record for China. It was known only from Norway and Denmark. The Chinese collection extends its distribution to Asia. The Chinese collection is identical with the epitype in TEF1 but with 2 bp differences for Hsp90 and 1 bp for LSU.

### Neotypification

#### Helvella taiyuanensis

Taxon classificationFungiPezizalesHelvellaceae

B. Liu, Du & J.Z. Cao, Acta Mycol. Sin. 4(4): 211, 1985

72A5BD1B-AD94-5CBC-8462-2F9222C29E07

Figure 4

##### Neotype is designated here.

CHINA. Shanxi Province, Lvliang City, Jiaocheng County, Guandishan National Forest Park, 37°54'25"N, 111°35'40"E, on the ground in mixed forest, 16 Jul 1987, Y.M. Li, HMAS 85689 (= MHSU 758).

##### Additional specimens examined.

CHINA. Beijing City, Mentougou District, Xiaolongmen National Forest Park, 39°58'2"N, 115°26'43"E, alt. 1100 m, on the ground in mixed forest, 4 Aug 2018, X.C. Wang et al. 11925, HMAS 254611. Hubei Province, Yichang City, Xingshan County, Longmenhe National Forest Park, 31°21'12"N, 110°30'40"E, on the ground, 23 Jul 2017, R. Wang & X. Zhang 420526MF0679, MCCNNU 6499, HMAS 279702. Yunnan Province, Diqing Tibetan Autonomous Prefecture, Dêqên County, Yunling Town, Meili Snow Mountain, 28°23'23"N, 98°47'49"E, alt. 3150 m, on the ground, 12 Aug 2016, Y. Li 920, HMAS 277500.

##### Notes.

This species was originally described, based on a single specimen collected by Y.M. Li from Taiyuan City, Shanxi Province in 1983 (Holotype: HBSU 2449, Liu et al. 1985). Unfortunately, the type specimen was destroyed by a fire in MHSU in 1984 (Cao 1988, Cao et al. 1990). To protect fungal collections after the fire, the remaining specimens, deposited in MHSU, were moved to HMAS. The neotype specimen HMAS 85689 was collected by the same collector as the type specimen of *H. taiyuanensis* and identified by one of the original authors J.Z. Cao (Cao 1988). Its detailed morphological characteristics are in accordance with the original description. We thus treat it as authentic material. As other specimens were neither cited in the protologue nor filed under this name, we thus designate HMAS 85689 as the neotype specimen of *H. taiyuanensis*.

**Figure 4.**
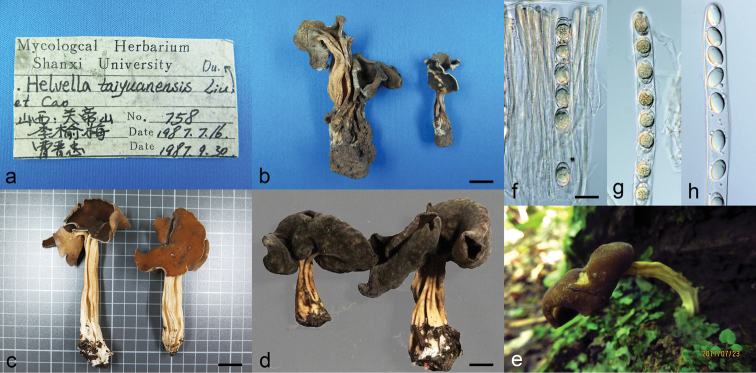
*Helvella taiyuanensis***a** specimen sheet (HMAS 85689) **b** mature apothecia when dry (HMAS 85689) **c** mature apothecia when fresh (HMAS 254611) **d** mature apothecia when fresh (HMAS 277500) **e** mature apothecium when fresh (HMAS 279702) **f–h** ascospores in ascus (**f, g**: HMAS 85689, **h**: HMAS 254611). Scale bars: 0.8 cm (**b, d**); 2 cm (**c**); 20 μm (**f**), applies to **g, h**.

*Helvella taiyuanensis* was once treated as a synonym of *H. solitaria* sensu Dissing (1966), based on morphological features (Cao 1988), but the molecular differences between them are clear in the multigene analysis (Figure 1). It should be a tenable species. The four specimens of the fungus examined are variable in colour of the hymenium and receptacle surface when dry or fresh, but stable in cupulate to saddle-shaped apothecia (Figure 4). Phylogenetic analyses indicate that they belong to the same species (Figures 1, 2 and Suppl. material 1: S1–S4) although minor sequence divergences exist amongst collections. The maximum sequence divergences amongst collections are 1 bp in Hsp90, 6 bp in ITS2, 3 bp in LSU and 7 bp in TEF1.

## Discussion

A total of about 28 rib-stiped cupulate species of *Helvella* and *Dissingia* have been reported in the world (Table 2) and 17 of them were investigated in this study. With the discovery of the three new species and one new record, 13 species were confirmed to be distributed in China. Amongst them, six are known only from China, five (*D. oblongispora*, *H. acetabulum*, *H. calycina*, *H. costifera* and *H. hyperborea*) are found in Europe and China and *D. confusa* and *H. solitaria* are widespread in Europe, Asia and North America. Amongst the Chinese helvellas, *H. acetabulum*, *H. costifera* and *H. taiyuanensis* show a relatively wide distribution range and occur in at least four provinces. However, *H. calycina*, *H. floriforma*, *H. sichuanensis*, *H. tianshanensis* and *H. tinta* were known only from a single locality. Eight species are in northwest China (Gansu, Qinghai, Shaanxi and Xinjiang), eight in the southwest (Sichuan and Yunnan) and seven in the north (Beijing, Inner Mongolia and Shanxi). However, the Chinese record of *H. leucomelaena* (≡ *D. leucomelaena*) (Teng 1963, Tai 1979, Zhuang 1998) is questionable since many specimens in HMAS, filed under that name, were based on misidentifications (Table 3).

As shown in the multigene phylogeny (Figure 1), three clades were formed amongst the investigated species. The cupulate *Helvella* taxa are clustered or mixed with the saddle-shaped ones. This gives the hint that the apothecial shape changed several times during the evolution. Clade 2, Clade 3 and *H. dryadophila* belong to the acetabulum-solitaria lineage (Skrede et al. 2017); however, this lineage was not herein supported due to joining of the non-cupulate species *H. crispa*. Clade 1 is in accordance with the costifera lineage (Skrede et al. 2017) with the addition of *H. tianshanensis*. Our results clearly support the separation of *Dissingia* from *Helvella* s. l. (Hansen et al. 2019).

Supplementary DNA barcodes are essential for delimitation of *Helvella* species. LSU is the most commonly used region for *Helvella* species identification (Nguyen et al. 2013, Landeros et al. 2015, Skrede et al. 2017). LSU is capable of distinguishing cupulate *Helvella* species (Suppl. material 1: Figure S3); whereas, its PCR amplification success rate is low (10/56), especially for specimens subject to long storage. A similar situation is witnessed in TEF1, which was suggested as a secondary barcode for fungi (Stielow et al. 2015). Although the primers for this region were reported working well on DNAs extracted from fresh materials, the amplifications from dried *Helvella* specimens were not easy (Skrede et al. 2017). The amplification success rate of TEF1 in our study was again low (15/56). Hsp90 was first applied to *Helvella* by Skrede et al. (2017) and is recommended due to its short sequence length, high amplification success rate, usefulness in species delimitation and its reasonable phylogenetic informative properties. It was successfully amplified from 53 of the 56 specimens studied and is able to distinguish all the involved species (Suppl. material 1: Figure S1). RPB2 was also applied in the recent studies (Skrede et al. 2017, Hansen et al. 2019), but did not work well since the amplicons of the newly designed primers, H_rpb2r2 and H_rpb2f, had a lower species resolution than that of Hsp90. The fragment is also too short to align with the existing sequences in GenBank.

ITS is recommended as the universal barcode for fungi (Schoch et al. 2012), which is applied widely to elucidate species diversity of the pezizalean ectomycorrhizae (Tedersoo et al. 2006, Healy et al. 2013, Hwang et al. 2015). However, very limited ITS sequences of cupulate *Helvella* species were available in GenBank. The trials of obtaining ITS amplicons, using the universal primers for many *Helvella* species, usually failed owing to primer mismatch (Skrede et al. 2017). The success rate of ITS amplification in our work was extremely low (2/56) upon using the primer pairs ITS5 and ITS4. Functional *Helvella*-specific ITS primers are expected to be developed. Our amplifications of the ITS2 region by the primers ITS3 and ITS4 reached a relative high success rate (47/56) with the tested species well separated (Suppl. material 1: Figure S2). We thus propose to use Hsp90 and ITS2 as supplementary DNA barcodes for rib-stiped cupulate species of *Helvella*.

## Supplementary Material

XML Treatment for Helvella acetabuloides

XML Treatment for Helvella sichuanensis

XML Treatment for Helvella tianshanensis

XML Treatment for Helvella calycina

XML Treatment for Helvella taiyuanensis

## Supplementary materials

Supplementary material 1 Figures S1–S4 Data type: phylogenetic data Explanation note: **Figure S1.** Maximum-likelihood phylogenetic tree of *Helvella* and its allies inferred from Hsp90 dataset. Bootstrap values ≥ 50% are indicated at nodes. **Figure S2.** Maximum-likelihood phylogenetic tree of *Helvella* and its allies inferred from ITS2 dataset. Bootstrap values ≥ 50% are indicated at nodes. **Figure S3.** Maximum-likelihood phylogenetic tree of *Helvella* and its allies inferred from LSU dataset. Bootstrap values ≥ 50% are indicated at nodes. **Figure S4.** Maximum-likelihood phylogenetic tree of *Helvella* and its allies inferred from TEF1 dataset. Bootstrap values ≥ 50% are indicated at nodes. https://binary.pensoft.net/file/352441This dataset is made available under the Open Database License (http://opendatacommons.org/licenses/odbl/1.0/). The Open Database License (ODbL) is a license agreement intended to allow users to freely share, modify, and use this Dataset while maintaining this same freedom for others, provided that the original source and author(s) are credited.Xin-Cun Wang, Tie-Zhi Liu, Shuang-Lin Chen, Yi Li, Wen-Ying Zhuang
