# Supplementary figures and images for: A four-locus phylogeny of rib-stiped cupulate species of Helvella (Helvellaceae, Pezizales) with discovery of three new species
Source: MycoKeys. 2019 Oct 31;60:45–67. doi: 10.3897/mycokeys.60.38186 (PMC6838221; doi:10.3897/mycokeys.60.38186)

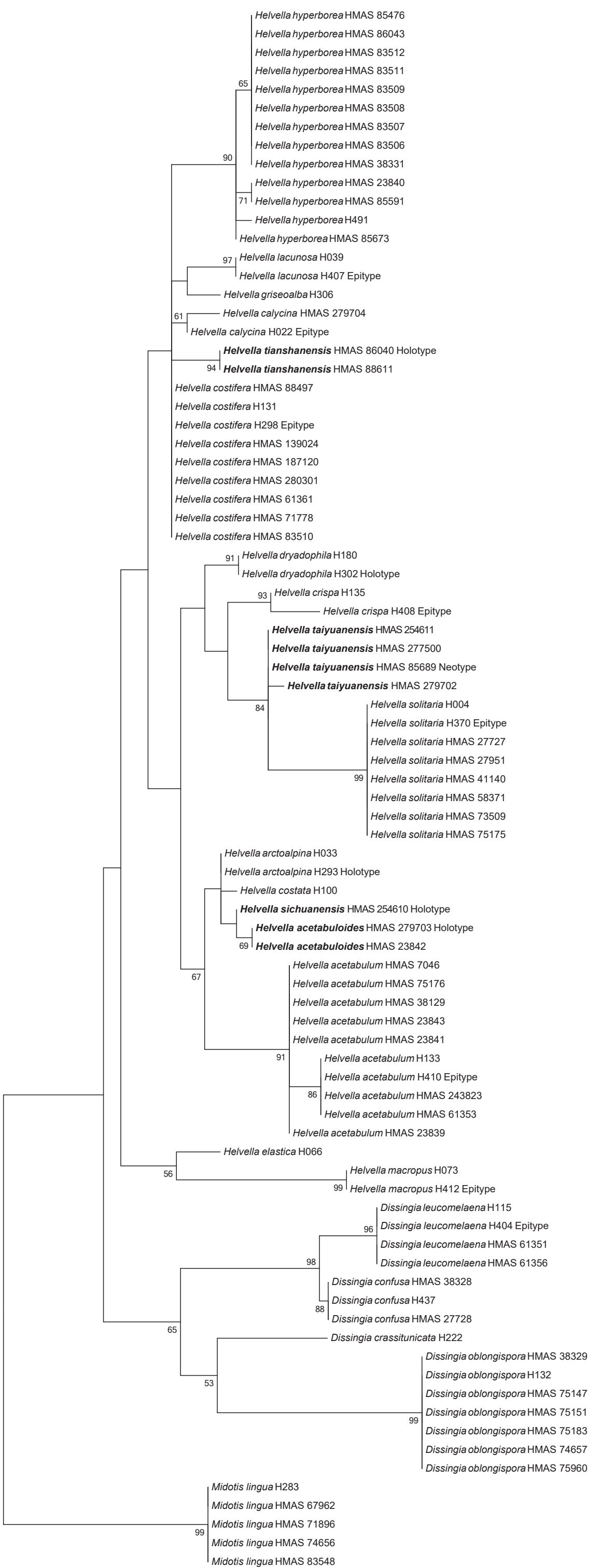

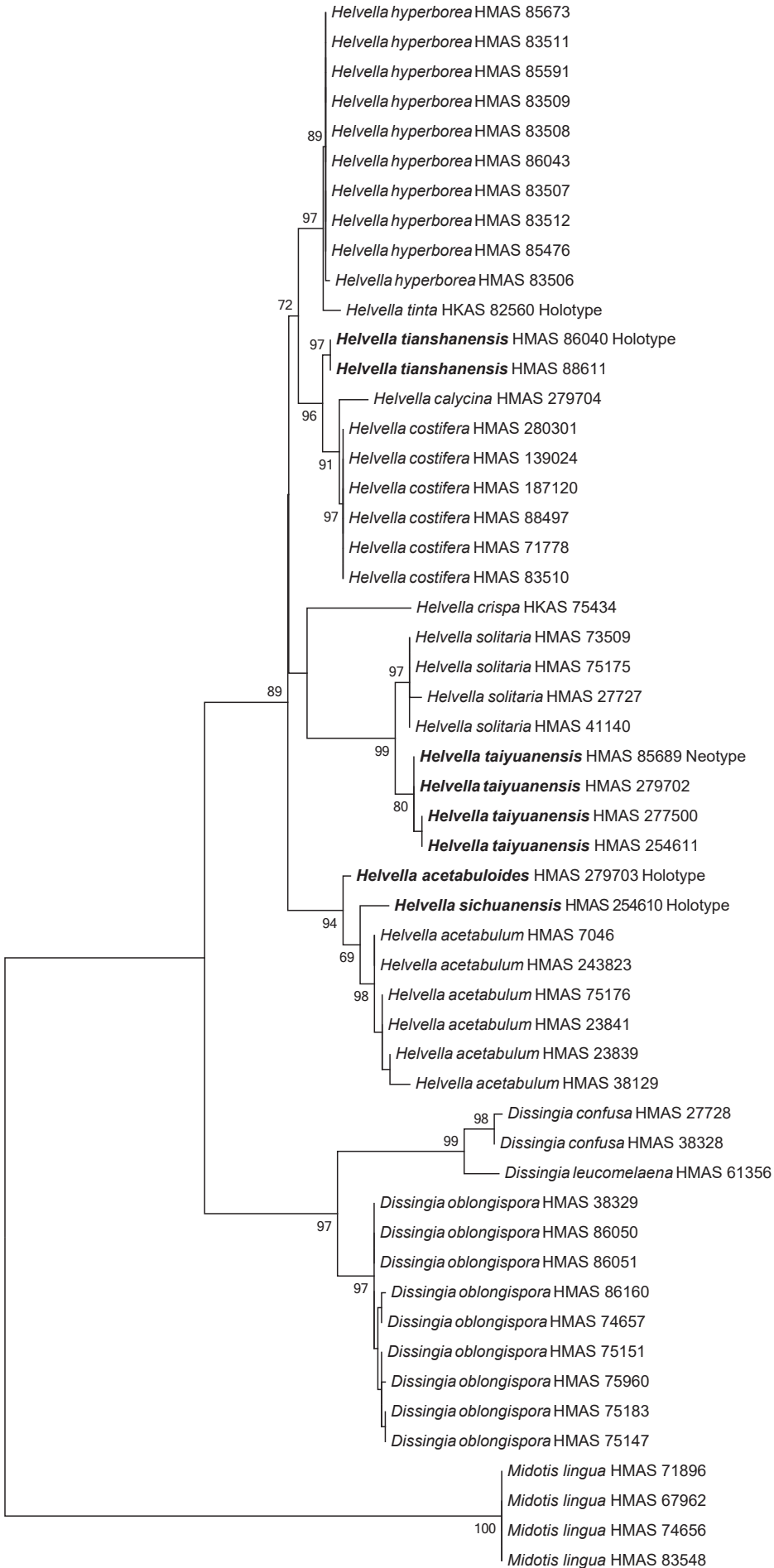

0.1

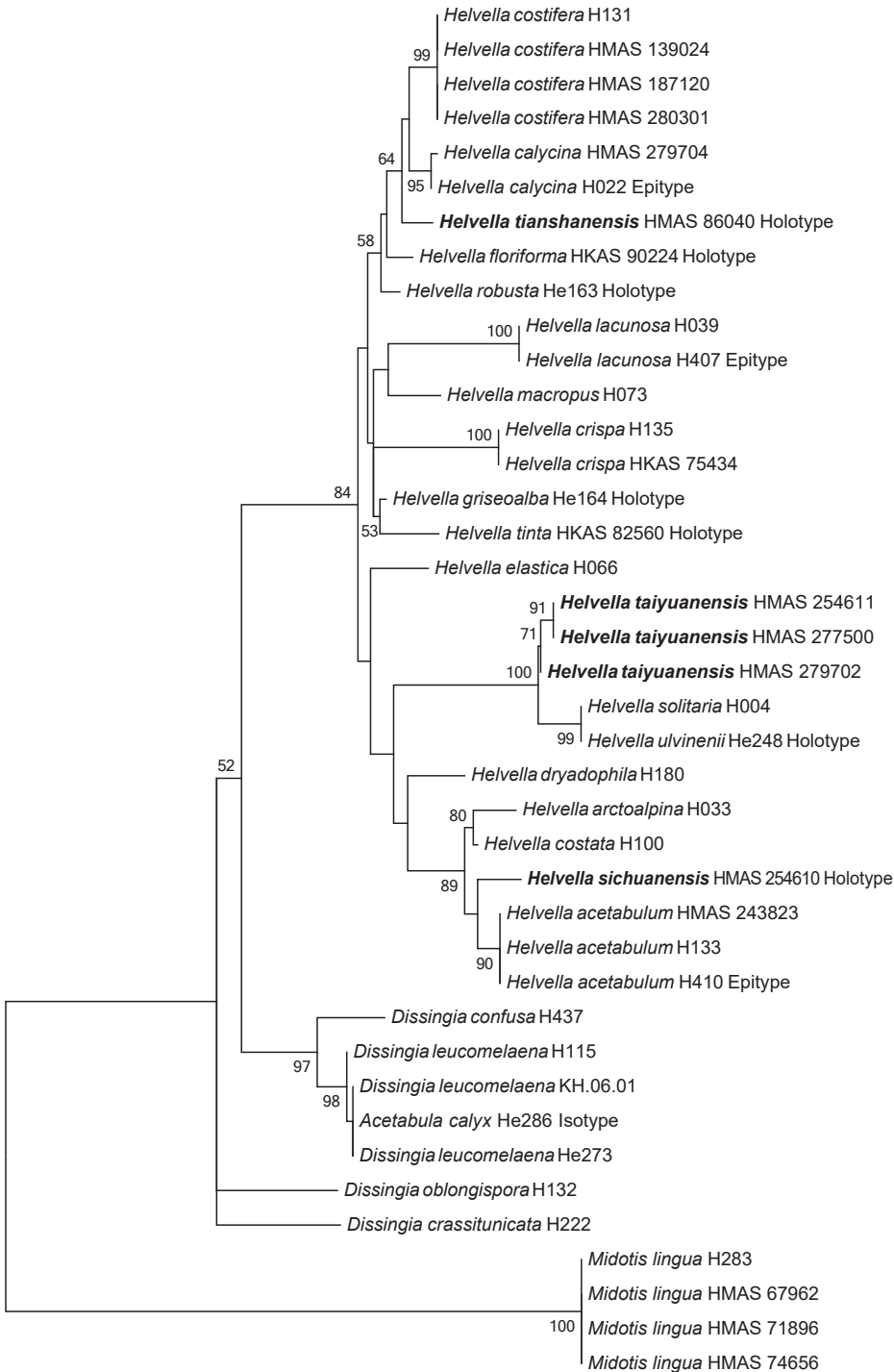

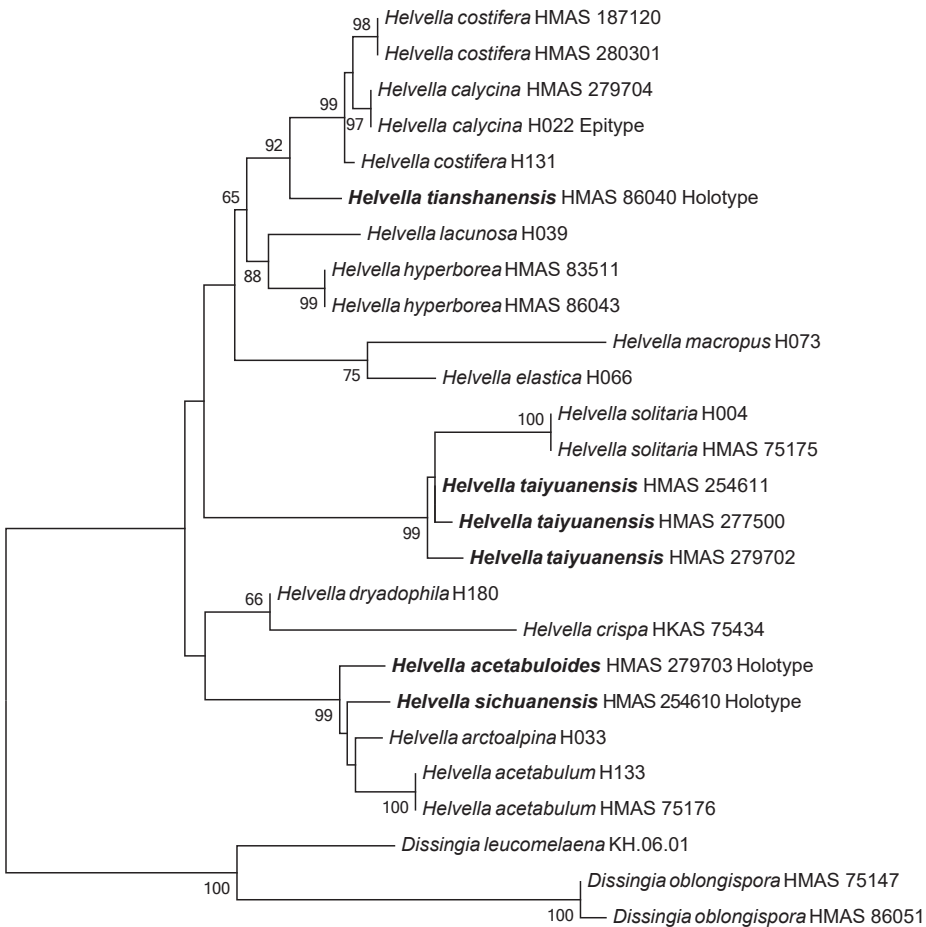

0.02

Supplement: Supplementary material 1 [file mycokeys-60-045-s001.pdf]
